# Generation of radially-polarized terahertz pulses for coupling into coaxial waveguides

**DOI:** 10.1038/srep38926

**Published:** 2016-12-12

**Authors:** Miguel Navarro-Cía, Jiang Wu, Huiyun Liu, Oleg Mitrofanov

**Affiliations:** 1 School of Physics and Astronomy, University of Birmingham, Birmingham B15 2TT, UK; 2 Department of Electronic and Electrical Engineering, University College London, Torrington Place, London WC1E 7JE, UK

## Abstract

Coaxial waveguides exhibit no dispersion and therefore can serve as an ideal channel for transmission of broadband THz pulses. Implementation of THz coaxial waveguide systems however requires THz beams with radially-polarized distribution. We demonstrate the launching of THz pulses into coaxial waveguides using the effect of THz pulse generation at semiconductor surfaces. We find that the radial transient photo-currents produced upon optical excitation of the surface at normal incidence radiate a THz pulse with the field distribution matching the mode of the coaxial waveguide. In this simple scheme, the optical excitation beam diameter controls the spatial profile of the generated radially-polarized THz pulse and allows us to achieve efficient coupling into the TEM waveguide mode in a hollow coaxial THz waveguide. The TEM quasi-single mode THz waveguide excitation and non-dispersive propagation of a short THz pulse is verified experimentally by time-resolved near-field mapping of the THz field at the waveguide output.

The coaxial waveguide exhibits no dispersion and no cut-off frequency for the TEM mode^1^. It is therefore an attractive transmission channel for broadband terahertz (THz) pulses^2^,^3^. The radial symmetry of this mode eliminates the need for maintaining the polarization direction required for most waveguides, and the wave confinement within the outer conductor ensures robust wave guiding compared to the surface Sommerfeld wave of metal wires^4^. Potential applications of THz coaxial waveguides and radially-polarized THz beams have led to the development of THz antennas with special radial electrodes^2^,^5^,^6^,^7^, and segmented nonlinear crystals with threefold rotational symmetry^8^,^9^. It was also demonstrated that radially-polarized THz waves can be generated directly on a polymer-coated metallic wire via optical rectification^10^. Such THz emitters however require complex fabrication processes or are not adaptable (i.e., once fabricated, they cannot produce a different polarization state). Diffractive optical elements in principle can synthesize any complex polarization state from a linearly polarized infrared pump beam^11^ (by controlling the wavefront photo-exciting a semiconductor) or THz beam^12^,^13^,^14^,^15^,^16^,^17^, but this approach involves a complex setup or has inherently bandwidth limitations and suffers from losses, respectively.

As an alternative solution for generation of radially-polarized THz pulses, one can consider physical processes that naturally produce radial THz fields. Optical rectification in a Cherenkov geometry with large velocity mismatch^18^ and air ionization by high-power ultrashort optical pulses^19^ generate such radially-polarized THz beams. However the ionization process also requires high-power amplified ultra-short laser pulses and the generated THz field in both schemes exhibits a conical wavefront. Recently, it was demonstrated that transient photo-currents excited on semiconductor surfaces by short (100 fs) optical pulses can have a large radial component due to the photo-generated charge density gradients^20^,^21^,^22^,^23^ and anisotropic conductivity^24^. This mechanism can provide efficiency, simplicity and tunability (i.e., dynamic control) in generation of THz beams with a tailored polarization state (e.g., radially-polarized THz beam) since the polarization state is governed by the shape of the optical beam and does not require bias. In this work, we exploit this radial photo-current mechanism for generation of radially-polarized THz pulses and efficient coupling into the TEM mode of a coaxial waveguide in a simple practical configuration (see Fig. 1(a,b)). The coupling scheme is inherently broadband, and optimal mode-matching between the radially-polarized THz pulses and the fundamental TEM mode is achieved for any coaxial waveguide size by adjusting the size of photoexcitation. Hence, coaxial waveguides of large diameters, which exhibit low attenuation for the TEM mode (indeed, the attenuation is minimum when the outer to inner diameter ratio is 3.6^1^), can be exploited for broadband dispersion-less waveguide transmission without the concern of higher order mode excitation.

Upon photoexcitation of a direct bandgap semiconductor by a short optical pulse, the photo-generated non-equilibrium clouds of electrons and holes undergo different dynamics leading to unbalanced transient spatial distribution of charge with corresponding dipole moments oriented in the surface plane^23^. The transient dipoles radiate pulses of THz waves with a spatial profile determined by the optical beam profile. A recent study of the field distribution of THz pulses generated at InGaAs surfaces photo-excited at normal incidence confirmed the significance of this mechanism^23^, which has been corroborated with Monte Carlo simulations^25^. For a Gaussian beam incident on the semiconductor surface, the transient dipole moments are arranged radially and the radiated THz pulse therefore is expected to be radially-polarized with the intensity profile in a doughnut shape^26^ similar to the TEM mode of the coaxial metallic waveguide.

## Experimental results: Waveforms and mode imaging

### InAs THz emission

**Figure 1:**
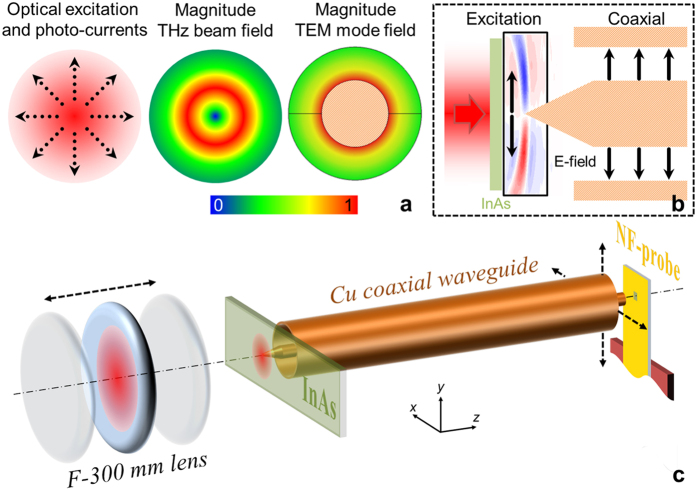
Generation of radially-polarized THz beam for coupling into the coaxial waveguide. (**a**) From left to right, optical excitation profile and photo-current (arrows) at the semiconductor surface; sketch of the electric field of the generated THz beam, |E_THz_|; and numerically-computed field distribution of the TEM mode, |E_TEM_|. (**b**) Cross-sectional view of the photoexcitation and coupling into the coaxial waveguide; the generated THz field distribution in the cross-section plane is displayed inside the rectangular solid frame. The distribution is obtained from a measured *xt*-map (see Fig. 2). (**c**) Sketch of the experimental setup. The 1 μm think indium arsenide (InAs) layer is sandwiched between a sapphire substrate for structural support and the 20 μm think GaAs layer for blocking the optical excitation.

A schematic diagram of the radially-polarized THz pulse generation and coupling into the coaxial waveguide is shown in Fig. 1(a,b). To produce the radially-polarized THz beam matching the coaxial waveguide TEM mode, we illuminated a semiconductor surface by a beam of 100 fs optical pulses of a Gaussian-like spatial distribution at normal incidence and varied the beam diameter (see Fig. 1(c) for a sketch). Details of the setup conditions can be found in Methods. InAs was chosen because of its narrow bandgap and high electron-to-hole mobility ratio required for creating transient dipole moments within the electron-hole cloud^27^. Growth details of the ~1 μm thick InAs can be found in Methods.

**Figure 2:**
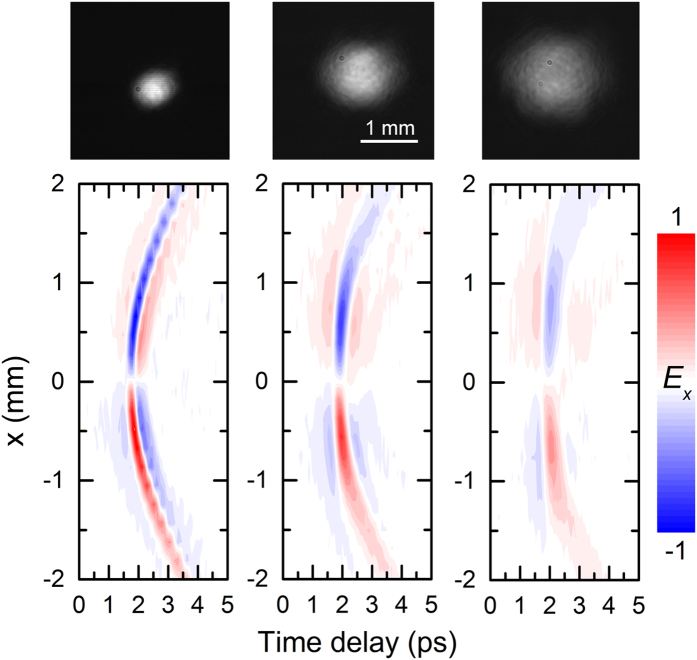
THz emission from the 1 μm thick InAs layer oriented normally to the incident optical beam. The space-time diagrams, *E*_*x*_(*x, t*), showing the change in the THz field wavefront as the focusing lens is scanned along *x* for three different sizes of optical beam waists (from left to right: 0.45, 1.05 and 1.48 mm). The corresponding images of the optical beams at the plane of the InAs surface taken with a CMOS camera are shown above the diagrams. The scale white bar is 1 mm.

Space-time maps of the THz electric field emitted from the InAs surface were measured by a stationary THz near-field probe^28^ positioned ~0.7 mm from the InAs surface, while the focusing lens was raster-scanned (which in turn raster-scans the excitation beam). Figure 2 shows the measured *xt*-maps, E_x_(*x,t*), for three different laser beam sizes (top panels). The maps show unambiguously the polarity inversion with respect to the optical axis characteristic of a radially-polarized beam. By using symmetry arguments, E_y_(*y*,*t*) must also show polarity inversion with respect to the optical axis. This radial polarization should ensure good overlap between the THz beam profile and a TEM mode. The smaller charge density gradient for larger laser beam size explains the observed amplitude decrease as the laser beam size increases.

### THz signal at the coaxial waveguide output

Next, a hollow coaxial waveguide with a 1 mm diameter copper-coated stainless steel rod (Fig. 1(c)), was placed within ~1 mm from the InAs layer. Further details of the coaxial waveguide fabrication can be found in Methods. The hollow space inside the waveguide minimizes transmission losses^1^,^2^ and ensures dispersionless propagation for the ideal TEM mode. We note that hollow waveguides allow achieving the lowest transmission loss at THz frequencies^29^. For instance, ref. 2 estimates the amplitude absorption coefficient of its coaxial waveguide SC-033/50 (Coax Co., Ltd. Yokohama, Japan) to be between 1.5 to 3 times larger than a hollow counterpart due to the Teflon filling. Ohmic losses in the metallic walls, scattering due to surface roughness and leakage through the split-block junction are the only loss mechanisms in the coaxial waveguide of this work. The theoretical attenuation of the TEM mode due to the dissipation in the smooth conductors (i.e., Ohmic losses)^1^ is under 25 dB/m (~0.058 cm^−1^) for the spectral window investigated.

**Figure 3:**
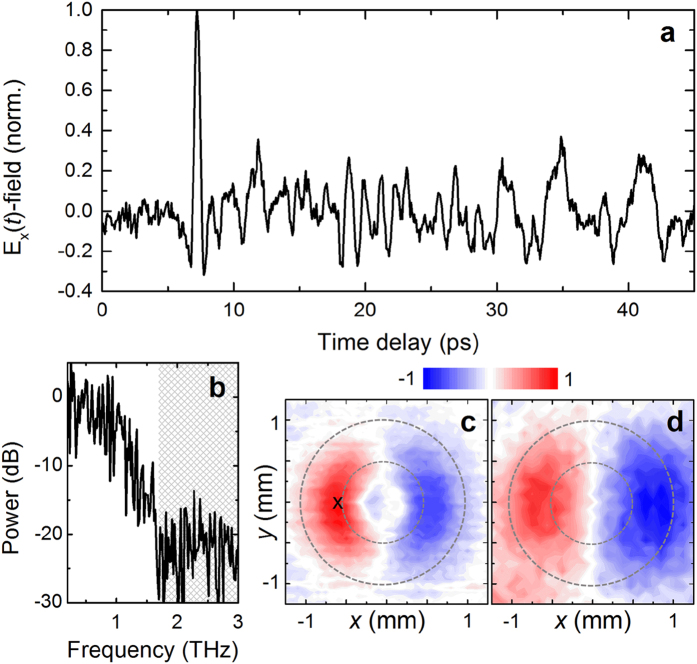
THz signal at the coaxial waveguide output. (**a**) THz pulse waveform detected after propagation through the 100 mm long 2 mm diameter hollow coaxial waveguide. (**b**) Normalized power spectra of the waveform in (**a**); the greyed area underlines the frequency range with the noise level. (**c**) Normalized near-field profile *E*_*x*_(*x, y*) at the waveguide output at the peak value of the corresponding waveform from (**a**) normalized to its maximum; the cross symbol shows the location where the waveform (**a**) was measured. (**d**) Normalized near-field profile *E*_*x*_(*x, y*) at the waveguide input at the peak of the THz pulse.

The THz pulse waveform and spectrum detected close to the inner conductor at the waveguide output are shown in Fig. 3(a,b) for the normal photoexcitation of the InAs surface. This waveform was taken after optimization of the coupling between the incident THz beam to the fundamental TEM waveguide mode (which, in turn, minimizes the excitation of higher-order modes), by changing the distance between the focusing lens and the InAs. The leading pulse in the waveform shows undistorted propagation through the waveguide. We assign this pulse to the TEM mode, since it is the only waveguide mode without dispersion. Indeed, the electric-field *xy*-map at the peak of the leading transmitted pulse reveals the TEM mode profile (Fig. 3(c)). The field distribution *E*_*x*_(*x, y*) at the output of the waveguide matches the incident field distribution measured approximately 1 mm away from the InAs crystal (Fig. 3(d)) for the optical excitation beam waist of 1.05 mm (middle column of Fig. 2). To optimize the coupling even further, the centre conductor of the coaxial waveguide at the input was tapered to ensure adiabatic shaping of the generated THz field into the TEM mode.

## Discussion

Within the THz generation model^23^, we expect that the profile of the generated THz pulse depends on the waist of the optical excitation beam. Specifically, the ring of maximum intensity in the doughnut shaped THz beam is expected to have a diameter similar to the optical beam waist. Therefore there must be an optimum beam waist that maximizes the coupling to the TEM mode. Using the energy density distribution of the fundamental TEM mode (Fig. 1(a)), we predict that the beam waist should be slightly larger than the inner conductor diameter.

**Figure 4:**
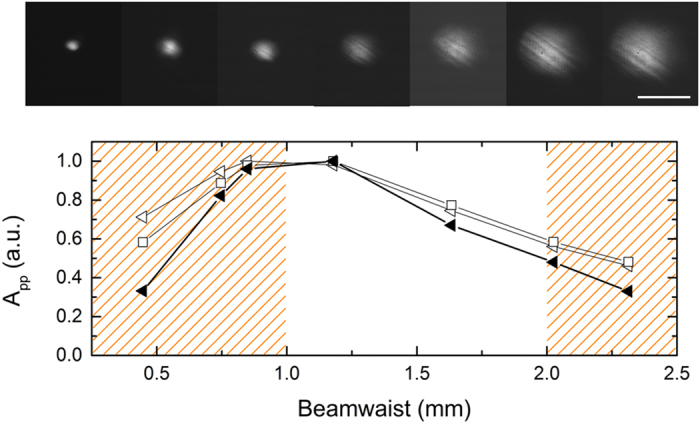
TEM mode peak-to-peak E-field amplitude in the coaxial waveguide vs. the optical beam waist. Experimental data (black triangles) and simulation data for a coaxial waveguide with (empty triangle) and without a tip (empty square). Dashed orange areas show the beam waist dimensions smaller than the inner conductor diameter and larger than the outer diameter of the coaxial waveguide clear aperture. The top strip shows images of the optical beams at the plane of the InAs surface taken with a CMOS camera. The scale bar is 2 mm.

Experimentally we varied the beam diameter by changing the distance between the focusing lens and the InAs sample. Figure 4 shows the variation of the measured peak-to-peak amplitude of the leading pulse (i.e., TEM mode). For small beam waists, the inner conductor blocks the radially-polarized THz excitation completely leading to poor coupling into the waveguide. As the optical beam diameter is increased, there is a rapid increase in the coupling efficiency, indicating that the size of the radially-polarized THz pulse becomes larger than the centre conductor diameter. The efficiency reaches a maximum and then decreases slowly. Eventually, the generated radially-polarized THz pulse is so large that the doughnut mode profile falls beyond the clear aperture of the coaxial waveguide, and thus, the coupling efficiency is small. We emphasize that, even though the smallest beam size yields the largest generated THz pulse amplitude (Fig. 2), it does not have good overlap with the waveguide TEM mode (because of the spatial mismatch), resulting in the low output amplitude of Fig. 4.

To support the experimental results shown in Fig. 4, we modelled in CST Microwave Studio^TM^ an analogous two-dimensional scenario. The technical details of the simulations can be found in Methods. The numerical amplitude peak-to-peak for varying beam diameter and a coaxial waveguide with and without the tapered tip is also plotted in Fig. 4 and shows a similar trend as the experimental data. The fall for smaller beam diameter is however less rapid than in the experiment, even for the coaxial waveguide without the tapered tip. The disagreement may indicate that saturation of THz emission happens for strongly focused optical beams.

**Figure 5:**
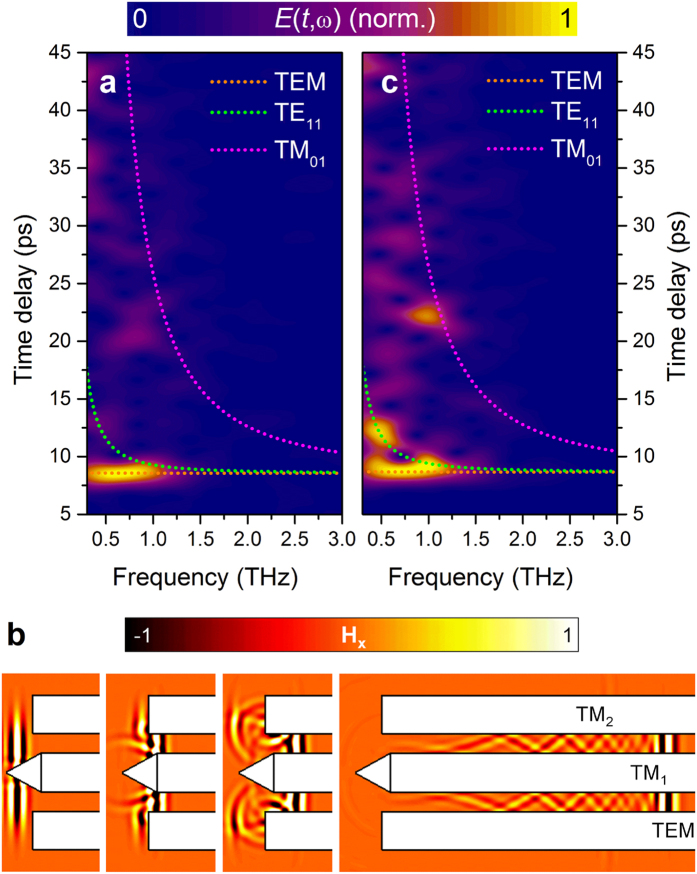
Experimental spectrograms at the coaxial waveguide output and *H*_*x*_(*y, z, t*)-field of a two-dimensional equivalent. (**a,c**) Time-frequency-maps of THz pulses at the output of the 100 mm long coaxial waveguide under radially-polarized (**a**) and linearly-polarized THz beam illumination (**c**). The numerically computed group delay for the TEM (dotted orange), the TE_11_ (dotted green) and TM_01_ modes (dotted magenta) are superimposed to facilitate the comparison. (**b**) Time evolution of the *H*_*x*_ field distribution on the *yz*-plane.

For practical applications of this scheme, it is also important to consider whether the incident beam excites other waveguide modes. The waveguide used in this work supports higher-order modes for the frequency range under investigation^1^. We examine the mode composition at the waveguide output by calculating the spectrograms, i.e., time-frequency-maps, of the measured output waveforms (see Fig. 5(a)). The waveform in Fig. 3 is analysed using 4.3 ps Hann windows (i.e., raised cosine windows). To assist in the discussion, the numerically-computed modal group delay for the first two waveguide modes (the fundamental TEM and the first higher-order TE_11_ modes^1^,^3^) along with the first higher-order mode with longitudinal polarization field component (TM_01_ mode^1^) are superimposed in the spectrograms; the eigenmode analysis follows the same methodology as in ref. 30 (see also Methods). Almost flat dispersion of spectral components at up to 1.5 THz is observed at *t* = 8 ps, the moment that corresponds to the leading THz pulse in Fig. 3(a). The flat dispersion is a signature of the TEM mode propagation. According to the modelling and assuming no leakage, the attenuation of the TEM mode ranges from 15 dB/m (~0.035 cm^−1^) at the low end to 45 dB/m (~0.104 cm^−1^) at the top end of the spectral window investigated^31^.

The numerical investigation of the coupling mechanism also reveals good excitation of the TEM mode (Fig. 5(b)). It also shows that the slight phase-front curvature of the emitted THz pulse along with the tapered tip cause the excitation of the TM_1_ (the two-dimensional equivalent of the TM_01_ coaxial waveguide mode) waveguide mode that follows the leading pulse (i.e., the TEM mode). The fact that the coupling efficiency to the TM_01_ mode is significantly lower than that of the TEM mode (for the optical beam waist = 1.18 mm) and that the attenuation of the TM_01_ mode is greater than the TEM mode (it ranges from 42 dB/m to 85 dB/m according to the modelling) prevents the detection of the TM_01_ mode after propagation in the 100 mm long waveguide in our experiment (Fig. 5(a)). Notice also that the arriving time of the low-frequency (<0.75 THz) components of the TM_01_ is beyond the scanned time window. For smaller optical beam waists, the coupling efficiency increases for the TM_1_ mode (it may even become the preferred excited mode) and also excitation of the TM_2_ and TM_3_ modes was observed in simulations.

To mitigate the influence of the optical focusing or the phase-front curvature of the emitted THz pulse on the coupling efficiency, an additional experiment with a different coaxial waveguide, an expanded infrared pump beam and a variable iris diaphragm that adjusts the beam size illuminating the InAs layer was carried out^31^. The results showed similar trend as Fig. 4, which serve as an independent validation of the efficient TEM mode excitation in the proposed scheme.

For comparison, we also consider coupling of a linearly-polarized THz beam into the same waveguide (Fig. 5(c)). To realize optimal coupling to the fundamental TEM mode, a linearly-polarized THz beam was shifted from the waveguide axis. Otherwise, only higher-order modes are excited since the coupling to the TEM mode is forbidden due to the waveguide symmetry^3^. The same InAs layer was used for generation of THz pulse, however, the surface was tilted at ~45 deg with respect to the optical pump beam to produce a linearly polarized THz beam of Gaussian profile^24^,^32^,^33^. The spectrogram in Fig. 5(c) confirms the TEM mode excitation. However significant energy is carried by a dispersive mode arriving at a later time. This mode is the TE_11_ mode as the numerical modelling reveals. Let us reinforce that for the normal to the surface photoexcitation, the trace associated to the TE_11_ mode is absent and even higher-order modes carry little energy compared to the main pulse, suggesting that quasi-single TEM mode propagation is obtained.

The difference in mode composition for the two excitation conditions is due to better matching of the incident THz beam to the TEM mode, which is radially-polarized, and thus, the E-field lines are symmetric with respect to the longitudinal waveguide axis. Meanwhile, the first higher-order mode is the TE_11_ mode with the E-field lines pointing in the same direction at the right and the left sides of the waveguide (i.e., anti-symmetric mode)^3^. If the illumination is focused on one side and is linearly-polarized as in the case of oblique incidence photoexcitation, there is no discrimination in excitation of the TEM and TE_11_ modes, and both will be excited. On the contrary, if the on-axis excitation is radially-polarized, the TE_11_ mode will not be excited due to the opposite symmetries of the excitation beam and the TE_11_ mode.

In conclusion, we exploit the effect of radially-polarized THz pulse generation via the radial transient photo-currents to achieve efficient coupling to the fundamental TEM mode of the coaxial waveguide. This method results in quasi-single mode dispersionless propagation of broadband THz pulses, which we achieve using a hollow coaxial THz waveguide. Unlike previously demonstrated excitation schemes for coaxial waveguides supporting a fundamental radially-polarized mode, the proposed approach requires no external bias and it can be adjusted easily for coaxial waveguides of various diameters. This work also suggests that the THz beam shape can be engineered by spatially shaping the infrared beam. The gradient of the photo-generated charge density has been linked to the amplitude of the emitted THz field^20^,^21^,^22^,^23^,^25^. Therefore the spatial profile and the polarization state of the THz pulse can be tailored by the spatial profile of the optical excitation. Since tailoring infrared radiation nowadays is routinely achieved with spatial light modulators, this method can provide a simple solution to the problem of realizing spatially structured THz beams^34^. Generation of arbitrary THz beam profiles, which can be reconfigured electronically, opens avenues for THz communications with space-division multiplexing and for more advanced THz signal processing, such as compressed sensing and cryptography with dynamic encoded patterns.

## Methods

### Fabrication: InAs and coaxial waveguide

**Figure 6:**
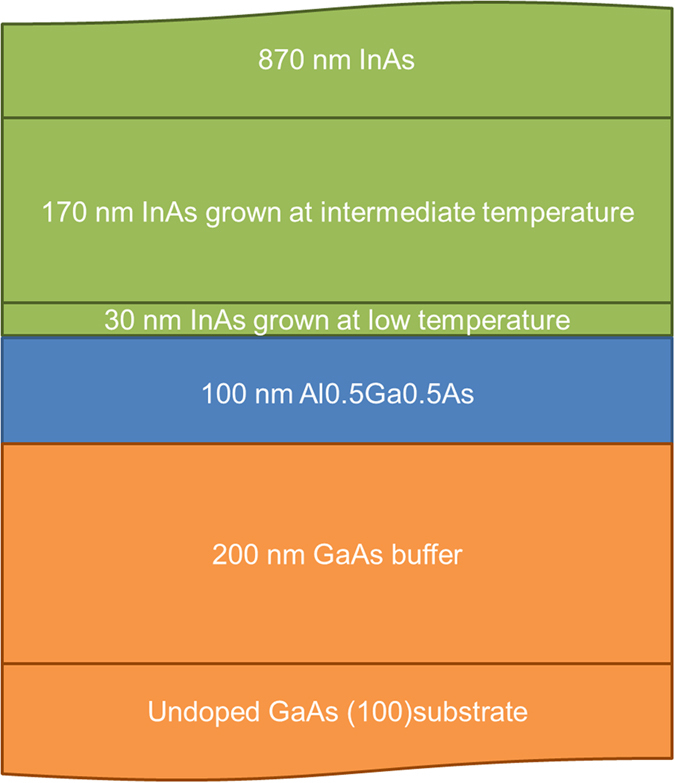
Details of the undoped InAs epilayer, not to scale.

A 1 μm thick undoped InAs epilayer was grown by molecular beam epitaxy on an undoped GaAs (100) substrate. The sample was bonded to a transparent 0.5 mm thick sapphire plate with epoxy and the GaAs substrate was mechanically thinned to a thickness of 20 μm. See Fig. 6 for details of the InAs epilayer.

The coaxial waveguide is composed of a 2 mm diameter 100 mm long cylindrical hollow waveguide machined in a copper split-block and a 1 mm diameter copper-coated stainless steel rod. The rod has a ~1 mm long linear taper of ~45 deg full angle and is suspended inside the cylindrical hollow waveguide with polyethylene caps at both ends of the copper block.

### Setup

The optical excitation of the InAs layer (see Fig. 1(c) for a sketch) and time-resolved detection of the THz waves were performed using 100 fs optical pulses with the central free-space wavelength of λ_0_ = 800 nm from a Ti:Sapphire mode-locked laser operating at the repetition rate of 78 MHz. The laser beam was first split into a pump and probe beam with a beam splitter.

The optical beam was modulated by a mechanical chopper at 2.7 kHz for low-noise lock-in detection and was focused with a lens (focal length of 300 mm). Adjusting the distance between this focusing lens and the InAs layer allowed us to change the beam size at the InAs surface from ~0.5 mm to 2.5 mm without changing the total optical power of ~400 mW while keeping the excitation below the THz emission saturation level for all sizes of the optical beam.

The probe beam passes through a step-scan delay stage to change the pump-probe delay times and gates a stationary sub-wavelength aperture (10 μm × 10 μm) integrated photo-conductive antenna that acts as a near-field probe^28^. This probe is positioned in close proximity to either the InAs surface or the coaxial waveguide output.

The output of the coaxial waveguide was mounted on an automated *xy*-translation stage for mapping the waveguide modes. The output waveguide fields were detected by the same above described THz near-field probe^28^, positioned at a short distance from the coaxial waveguide (less than 1 mm). The spectrum was computed via Fourier transform of the measured waveform.

### Two-dimensional simulation

The transient solver of CST Microwave Studio^TM^ is used to model the emission and waveguide in-coupling mechanism. To alleviate computational effort, a two-dimensional scenario translationally invariant along *x*-direction is considered together with a horizontal (y = 0) magnetic mirror plane. We model the transient in-plane photo-current with 50 solver-defined current sources of 10 μm length vertically-oriented and distributed along *y*-direction with a centre-to-centre separation of 50 μm. The current amplitude of each source corresponds to the derivative of the optical excitation intensity profile (i.e., Gaussian profile) at such position. The current transient is defined as a short pulse to cover the frequency range from 0.25 to 1.5 THz that matches the frequency content of the wave detected experimentally and predicted analytically via Monte Carlo simulations^25^. The dimensions of the coaxial waveguide model are those previously described, but the length that is fixed to 20 mm. A hexahedral mesh with varying cell size between 5 to 20 μm is used to map accurately the geometry, particularly the tip. The output waveform is detected with a point probe at the middle point between the inner and outer conductors. To avoid reflections from the truncation of the waveguide, the solver-defined open boundary condition is used at the end of the waveguide. This open boundary condition makes the model effectively to mimic a semi-infinite coaxial waveguide. The *H*_*x*_-field distribution on the *yz*-plane is recorded as a function of time to oversee the in-coupling mechanism.

### Eigenmode simulation

In order to model the experiment accurately, we calculate the eigenmodes and the corresponding eigenvalues (dispersion diagram) of the coaxial waveguide numerically with the 2D frequency-domain eigenmode solver of CST Microwave Studio^TM^. An effective dispersive conductivity calculated via the Hammerstad-Jensen approach that takes into account the roughness of the metal (assumed to be 5 μm RMS for this study) is used to model copper. The simulated geometry consists of an ideal loss-free air ring of internal and external diameter of 1 and 2 mm, respectively. Given the twofold symmetry of the waveguide and assuming perfectly aligned symmetric excitation, a vertical (x = 0) electric and a horizontal (y = 0) magnetic mirror planes are applied to consider only a quarter of the waveguide cross-section. An adaptive tetrahedral discretization with maximum edge length of 10 μm is used to match accurately the circular cross-sectional area of the waveguide.

## Additional Information

**How to cite this article**: Navarro-Cía, M. *et al*. Generation of radially-polarized terahertz pulses for coupling into coaxial waveguides. *Sci. Rep.* **6**, 38926; doi: 10.1038/srep38926 (2016).

**Publisher's note:** Springer Nature remains neutral with regard to jurisdictional claims in published maps and institutional affiliations.
